# CD1c-Related DCs that Express CD207/Langerin, but Are Distinguishable from Langerhans Cells, Are Consistently Present in Human Tonsils

**DOI:** 10.3389/fimmu.2016.00197

**Published:** 2016-05-25

**Authors:** Anne De Monte, Charles-Vivien Olivieri, Sébastien Vitale, Sonanda Bailleux, Laurent Castillo, Valérie Giordanengo, Janet L. Maryanski, Elodie Segura, Alain Doglio

**Affiliations:** ^1^Laboratory MICORALIS EA7354, Faculté de chirurgie dentaire, Université Nice-Sophia-Antipolis, Nice, France; ^2^Laboratory Unité de Thérapie Cellulaire et Génique (UTCG), Centre Hospitalier Universitaire de Nice, Hôpital Pasteur, Nice, France; ^3^Laboratory of Virology, Centre Hospitalier Universitaire de Nice, Hôpital l’Archet, Nice, France; ^4^Department of Pediatric Otorhinolaryngology, Hôpitaux pédiatriques de Nice CHU-Lenval, Nice, France; ^5^Department of Otorhinolaryngology, Institut Universitaire de la Face et du Cou, Nice, France; ^6^INSERM U932, Institut Curie, Paris, France

**Keywords:** tonsil, dendritic cells, antigen-presenting cells, langerin, CD207, Langerhans cells, inflammatory dendritic cells, CD1c myeloid dendritic cells

## Abstract

Several subsets of dendritic cells (DCs) are present in the oropharyngeal tonsillar tissues and are thought to behave as major actors in development and regulation of immunity by acting as a first line of recognition for airborne and alimentary antigens. We previously discovered in human adult tonsils infected with Epstein–Barr virus (EBV), a subset of DCs that expressed langerin/CD207, a lectin usually recognized as a hallmark of epidermal Langerhans cells (LCs). In the present study, we analyzed the content of several child and adult tonsils in order to characterize in more detail the phenotype of these tonsillar CD207-expressing DCs (tCD207 DCs) and to compare it with that of other human DC subsets. We showed that all the human tonsils studied (*n* = 12) contained significant proportions of tCD207 DCs among tonsillar cells expressing HLA-DR. Moreover, the presence of tCD207 DCs in tonsils from young children free of EBV infection indicated that these cells could be established early in the tonsil independently of EBV infection. We also showed that tCD207 DCs, that were found mainly located within the tonsillar lymphoid stroma, were distinguishable from LCs by the level of expression of CD1a and EpCAM, and also from human inflammatory DCs by the lack of CD1a, CD206, and CD14 expression. Detailed analysis of cell surface DC markers showed that tCD207 DCs were unrelated to CD141^+^ DCs or macrophages, but defined a subtype of tonsillar DCs closely related to myeloid resident CD1c DCs. Since it was established that blood CD1c myeloid DCs exhibit plasticity and are capable of expressing CD207 notably in the presence of inflammatory cytokines, it is tempting to speculate that CD207^+^ CD1c^+^ DCs may play a specific immune role.

## Introduction

The Waldeyer’s ring is a ring of lymphoid tissue that circles the pharynx at the entrance of the aerodigestive tract. This mucosa-associated lymphoid tissue notably contains the palatine tonsils that form dense compact bodies of lymphoid tissue and the adenoids, tubar, and lingual tonsil. The Waldeyer’s ring grows throughout childhood until the age of 11 years before steadily declining with age (1, 2). These oropharyngeal immune tissues are the first organs in the lymphatic system that analyze and react to airborne and alimentary products with the basic function to produce antibodies to common environmental antigens. Dendritic cells (DCs) that colonize tonsils are thus thought to play a pivotal role in controlling development of immunity since these professional antigen-presenting cells (APCs) have the capacity to induce a primary immune response in naïve T lymphocytes, and to play a pivotal immune role in regulating innate and adaptive immune responses (3–5).

Usually human blood DCs have been divided into two main groups: plasmacytoid DCs (pDCs) and “myeloid” or “classical” DCs (cDCs). The cDCs can be further separated into two subsets that are usually referred to as BDCA1/CD1c^+^ DCs and BDCA3/CD141^+^ DCs (6), which are also found in all lymphoid organs and represent resident DCs (5, 7–9). Although pDCs and cDCs subsets have been widely characterized in human tonsils (3–5, 10, 11), additional tonsillar DC subsets have also been identified (12, 13). We previously discovered in adult human tonsil a new type of human DC expressing the type II transmembrane C-type lectin receptor langerin (CD207) (12). These tonsillar CD207-expressing DCs (tCD207 DCs) abundantly infiltrated Epstein–Barr virus (EBV)-infected areas in tonsil and were also commonly encountered in EBV-infected tumors (i.e., nasopharyngeal carcinoma and Hodgkin’s lymphoma) (12). Langerin/CD207 typically characterizes Langerhans cells (LCs) that represent migratory DCs present in the epidermis and form a distinct cell lineage (14–16). Interestingly, Bigley and co-workers (17) have recently shown that CD207 was also expressed on human DCs isolated from dermis, lung, liver, tonsil, and lymphoid tissue. These CD207-expressing DCs were shown to be distinguishable from conventional LCs by the expression level of CD1a, EpCAM, CD11b, CD11c, CD13, and CD33, and were proposed to be closely related to the CD1c^+^ DCs. Moreover, transcriptomic analysis has also established that CD207 can be expressed by human “inflammatory” DCs (infDCs), a population of monocyte-derived DCs with a distinct phenotype that is commonly encountered in inflamed tissues (18, 19). In this report, we set out to further characterize the tCD207 DCs we previously discovered in tonsils, and we found evidence of CD207 expression by CD1c^+^ myeloid DCs.

## Materials and Methods

### Human Specimen Collection

Palatine tonsils were collected from 10 healthy children (aged from 1 to 11 years old, average 4.5 years, and median 3 years) undergoing routine tonsillectomy (Table 1). Seven children had surgery for tonsillar hypertrophy inducing obstructive sleep apnea syndrome and three for recurrent tonsillitis. In addition, tonsils from two healthy adults were also collected for recurrent tonsillitis. Tonsils were directly obtained at the operating room of the pediatric and adult otorhinolaryngology departments of Nice hospitals and processed extemporaneously for cell dissociation. This study was carried out with approval from the national ethical committee, “Comité de Protection des Personnes” department “Sud-Méditerranée V,” with written informed consent from all donors or parental donors for children. All subjects gave written informed consent in accordance with the Declaration of Helsinki.

**Table 1:** **Characteristics of the study population**.

Patient^a^	Age	Gender	Tonsillectomy indication	tCD207 DCs^b^	CD1c DCs^b^	CD141/Clec9A DCs^b^	pDCs^b^	Macrophages^b^	EBV status^c^
Child 1	1	M	Tonsillar hypertrophy	1.7	7.1	3.0	84.5	2.3	Negative
Child 2	3	M	Tonsillar hypertrophy	1.3	5.9	5.2	75.4	3.3	Positive
Child 3	3	M	Tonsillar hypertrophy	1.5	2.8	2.5	70.7	13.3	Negative
Child 4	3	M	Tonsillar hypertrophy	4.0	8.0	8.8	79.6	2.7	Negative
Child 5	3	F	Recurrent tonsillitis	5.6	13.1	6.0	58.8	6.5	Positive
Child 6	4	M	Tonsillar hypertrophy	0.6	3.2	2.6	88.6	1.2	Positive
Child 7	4	M	Recurrent tonsillitis	1.5	4.3	2.4	85.9	0.8	Positive
Child 8	4	M	Tonsillar hypertrophy	3.7	8.3	5.8	58.5	2.8	Positive
Child 9	9	F	Recurrent tonsillitis	4.7	12.0	2.8	63.2	5.8	Positive
Child 10	11	F	Tonsillar hypertrophy	7.5	10.7	0.8	78.1	1.5	Positive
Mean (SD) in child’s group				3.2 (2.2)	7.5 (3.6)	4.0 (2.4)	74.3 (11.1)	4.0 (3.7)	N/A
Adult 11	49	M	Recurrent tonsillitis	6.9	18.7	2.0	57.6	7.5	Positive
Adult 12	52	M	Recurrent tonsillitis	7.8	18.1	4.9	55.8	2.3	Positive
Mean (SD) in adult’s group				7.3 (0.6)	18.4 (0.4)	3.4 (2.0)	56.7 (1.3)	4.9 (3.7)	N/A

*^a^Data are provided per subject*.

**Figure 1:**
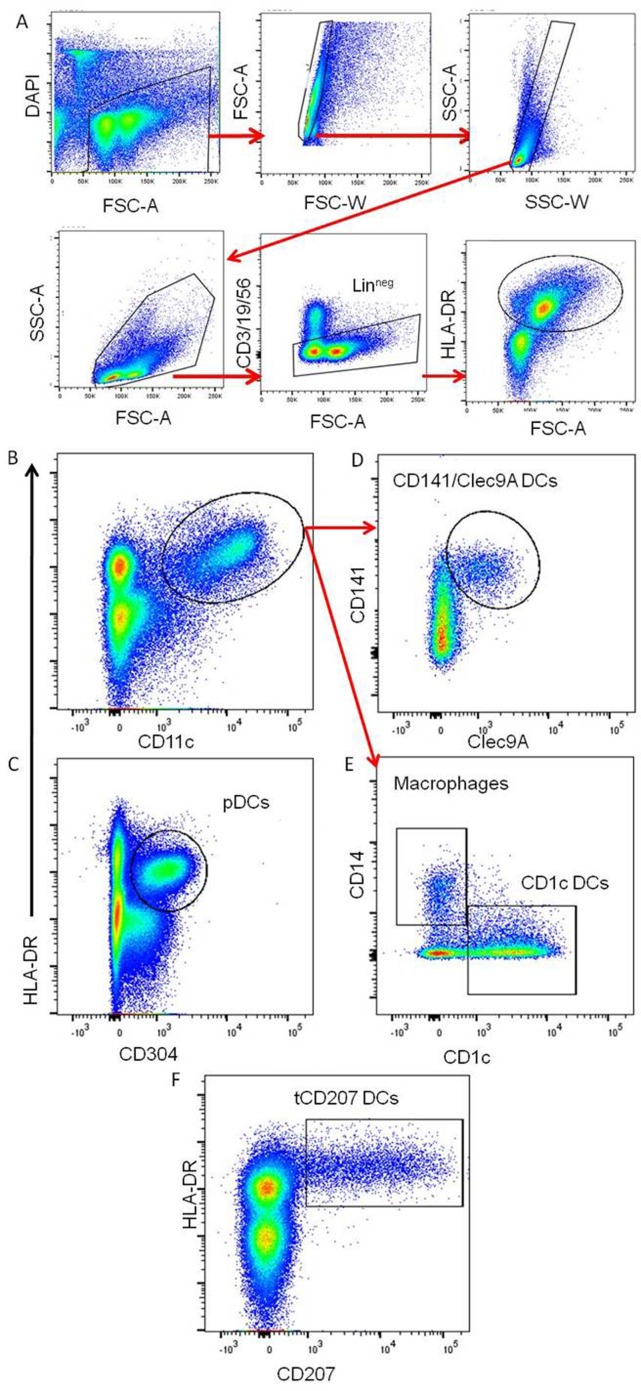
**Identification of different DC subsets in tonsils**. Representative gating strategy to define the main tonsillar DC subsets. **(A)** Gating strategy to identify viable Lin^neg^ HLA-DR^+^ tonsillar mononuclear cells. **(B,C)** The panels represent tonsil mononuclear cells gated as Lin^neg^ and analyzed for HLA-DR, CD11c, and CD304. **(D,E)** The panels show gated Lin^neg^ HLA-DR^+^ CD11c^+^ cells (red arrows) analyzed for expression of CD141, clec9A, CD1c, and CD14. The main tonsillar subsets are indicated. **(F)** Plot represents gated Lin^neg^ cells analyzed for expression of HLA-DR and CD207. These data are representative of 12 independent experiments (10 children and 2 adults).

*^b^Flow cytometric data are presented as % of Lin^neg^HLA-DR^+^ cells gated as shown in Figure 1. Each DC population was defined as follows: tCD207 DCs are HLA-DR^+^ CD207^+^; CD1c DCs are HLA-DR^+^ CD11c^+^ CD1c^+^; CD141/Clec9A DCs are HLA-DR^+^ CD11c^+^ CD141^+^ Clec9A^+^; pDCs are HLA-DR^+^ CD304^+^; and macrophages are HLA-DR^+^ CD11c^+^ CD14^+^*.

*^c^EBV status was defined for each child by EBV serology interpretation and for adults by EBV DNA detection*.

*N/A, non-applicable*.

### Tonsillar Cell Isolation and Flow Cytometric Analysis

Release of mononuclear cells from tonsil stroma was performed as previously described (3). The tonsils were kept in RPMI 1640 medium (Gibco^®^), then cut into small fragments, and incubated for 30 min with constant stirring in the presence of dissociation enzymes [Liberase TL (Roche) 0.1 mg/ml, DNase (Roche) 0.1 mg/ml]. Mechanical dissociation of tissues was also achieved with the gentleMACS™ dissociator (Miltenyi Biotec) used before and after enzyme digestion. After filtration through a 40-μM filter (cell strainers, BD Biosciences), tonsillar mononuclear cells were collected by density gradient centrifugation (Lymhocytes Separation Medium, Eurobio) and depleted of T cells and B cells by immunomagnetic selection (CD3 and CD19 microbeads, Miltenyi Biotec). DC-enriched samples were then stained with fluorochrome-conjugated mouse monoclonal antibodies for the detection of cell surface markers as follows: HLA-DR BV605 (BioLegend), CD3 FITC or APC (BD Biosciences), CD19 FITC or PE (BD Biosciences), CD56 FITC (BD Biosciences), CD207 PE-Vio770 or APC-Vio770 (Miltenyi Biotec), CD1a PerCp-Cy5.5 (BioLegend), CD11c APC (eBioscience), CD141 APC-Vio770 (Miltenyi Biotec), Clec9A PE (BioLegend), CD1c APC or PE-Cy7 (eBioscience), CD14 PE or FITC (eBioscience), CD206 APC (BD Biosciences), FcERI PE (BioLegend), CD304 APC (Miltenyi Biotec), and CD45 PE-Cy7 (BD Biosciences). Non-specific fixation of antibodies to human Fc receptors was blocked (Human Fc Receptor TruStain FCX™ Blocking Solution, BioLegend). Non-viable cells were excluded by DAPI staining (BD Biosciences). Fluorescence-minus-one (FMO) controls were performed to define background fluorescence for each marker. Multiparameter fluorescence-activated cell sorting (FACS) analysis was performed using a FACS Canto II (BD Biosciences), and data were analyzed with FlowJo software.

### EBV Infection Status

Epstein–Barr virus infection status was defined by serology for children. Serological tests for antibodies specific for EBV antigens were used to define infection status [viral capsid antigen (VCA) IgG, VCA IgM, and EBV nuclear antigen (EBNA) IgG] (Liaison analyzer, Diasorin). The presence of VCA IgG and VCA IgM, or VCA IgG and EBNA IgG indicated positive infection, and the absence of VCA IgG, EBNA IgG, and VCA IgM characterized uninfected children. For the two adults, the EBV infection status was defined by the EBV DNA detection in tonsils using standard PCR (EBV R-gene, Argène, BioMérieux).

### Immunohistochemical Studies

Fragments from each tonsil were fixed in formalin, embedded in paraffin, and processed for histological analyses. Five-micron sections of formalin-fixed paraffin-embedded tissues were deparaffinized, rehydrated, and stained with hematoxylin and eosin (H&E). Tissue sections were treated for heat-induced epitope retrieval using a pre-heated solution EDTA pH 9 at 97°C, and then non-specific staining was blocked with peroxidase-blocking reagent (EnVision™ FLEX Peroxidase-Blocking Reagent, Dako) and incubated with CD207 mouse monoclonal antibody (Bio SB) diluted 1:50 in antibody diluent (EnVision™ FLEX Antibody Diluent, K8006, Dako). Immunohistochemical studies (IHC) were performed using the EnVision™ FLEX Mini Kit detection system (K8023, Dako).

### Statistical Analysis

Statistical analysis was carried out with Easy Med Stat© (www.easy-med-stat.com). When evaluating differences in continued variables between two unpaired small groups, the Mann–Whitney *U* test was performed. When an association between two small groups was researched, the Fisher test was used, and correlation coefficients were calculated using Spearman’s rank method. *p* values of <0.05 were considered significant.

## Results

### Consistent Presence of Langerin/CD207-Expressing DCs in Human Tonsils

This study was focused on tonsils from 10 children and from 2 adults as summarized in Table 1. The DC-enriched tonsillar fractions from all tonsils were analyzed by FACS gated on lineage negative (Lin^neg^: CD19^−^CD3^−^CD56^−^) mononuclear cells expressing HLA-DR (Lin^neg^ HLA-DR^+^) (Figure 1A). Accordingly to previous work (5), different subsets of HLA-DR^+^ DCs were identified within tonsils (Figures 1B–E). The pDCs (HLA-DR^+^ CD304^+^) (Figure 1C) were the most abundant tonsillar DCs with a frequency ranging around 74.3% (±11.1) of the tonsillar HLA-DR^+^ cells in the child group (*n* = 10) and 56.7% (±1.3) in adults (*n* = 2) (Table 1). Classical myeloid DC subsets, such as CD1c DCs (Figure 1E), CD141/Clec9A DCs (Figure 1D), and macrophages (Figure 1E), were also consistently detected and represented around 7.5% (±3.6), 4.0% (±2.4), and 4.0% (±3.7), respectively, in the child group (*n* = 10) and 18.4 (±0.4), 3.4 (±2.0), and 4.9 (±3.7), respectively, in adults (*n* = 2). Moreover, all tonsils also contained CD207-expressing HLA-DR^+^ cells (tCD207 DCs) (Figure 1F) with an average frequency of 3.2% (±2.2) in children and 7.3% (±0.6) in adults (Table 1). Variable levels (0.6–7.5%) of tCD207 DCs were found among the child tonsils studied but intriguingly, the oldest child (child 10 and 11 years) had the highest level of tCD207 DCs (7.5%), similar to the level found for the two adult tonsils (6.9 and 7.8%) analyzed (Table 1). As shown in Table 1, independently of the EBV infection status, tCD207 DCs were always detected in tonsils and we did not find any correlation between tCD207 DC frequency and EBV infection status. Their consistent presence in child and adult tonsils suggests that tCD207DCs may play an important role in immune functioning of the oropharyngeal lymphoid tissues.

### tCD207 DCs Are Distinct from LCs and infDCs and Represent a Subset of Resident CD1c^+^ DCs

**Figure 2:**
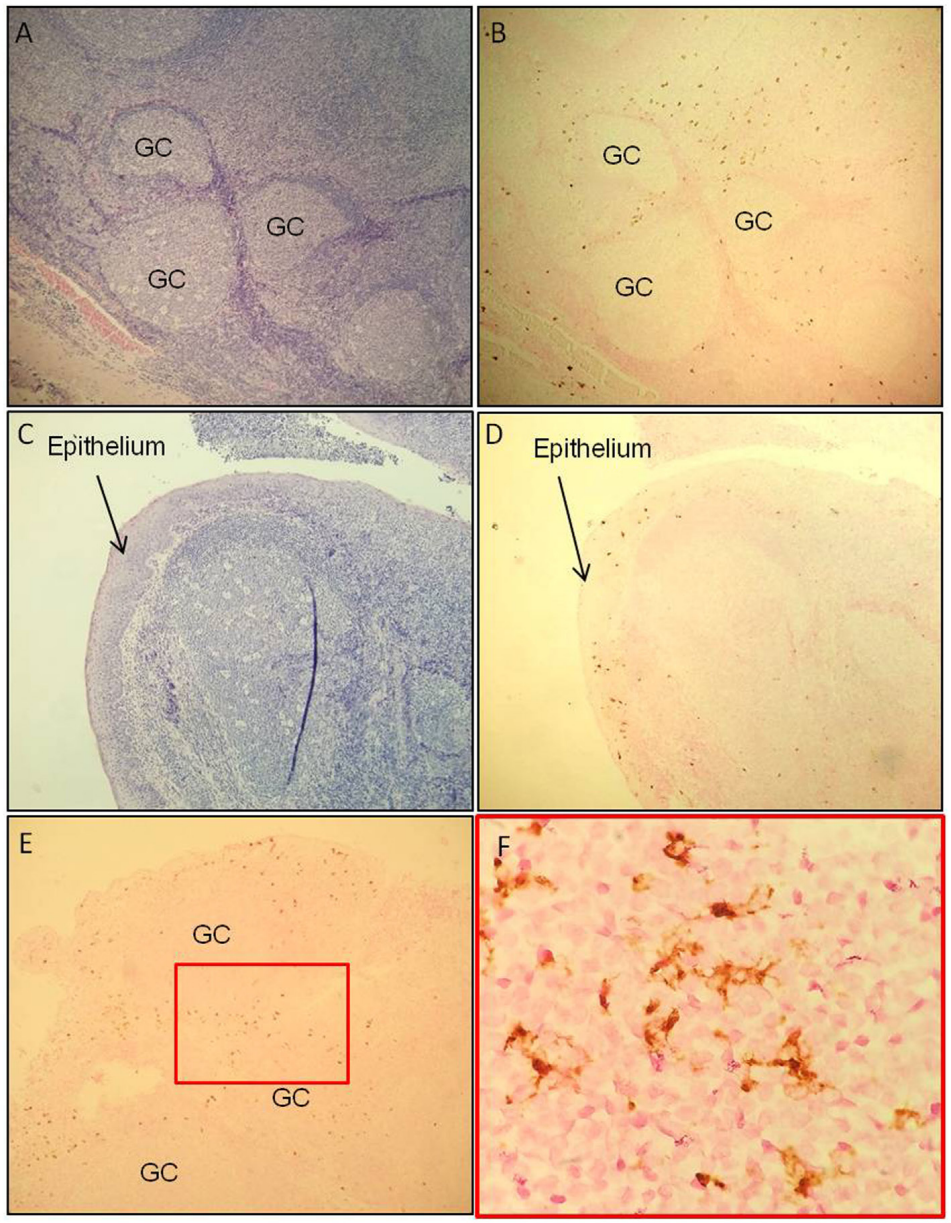
**tCD207 DCs are located in tonsillar lymphoid stroma**. **(A,C)** H&E and **(B,D)** CD207 immunohistochemical staining (brown) of a tonsillar paraffin-embedded tissue sections (low magnification 10×). These analyses were performed on 10 children tonsillar paraffin-embedded tissue sections and 2 adults. The tCD207 DCs are located **(A,B)** outside the germinatif centers (GC) and **(C,D)** in the epithelium. **(E,F)** Characteristic DC morphology, high magnification of CD207 immunohistochemical staining, 50× **(E)** and 100× **(F)**.

**Figure 3:**
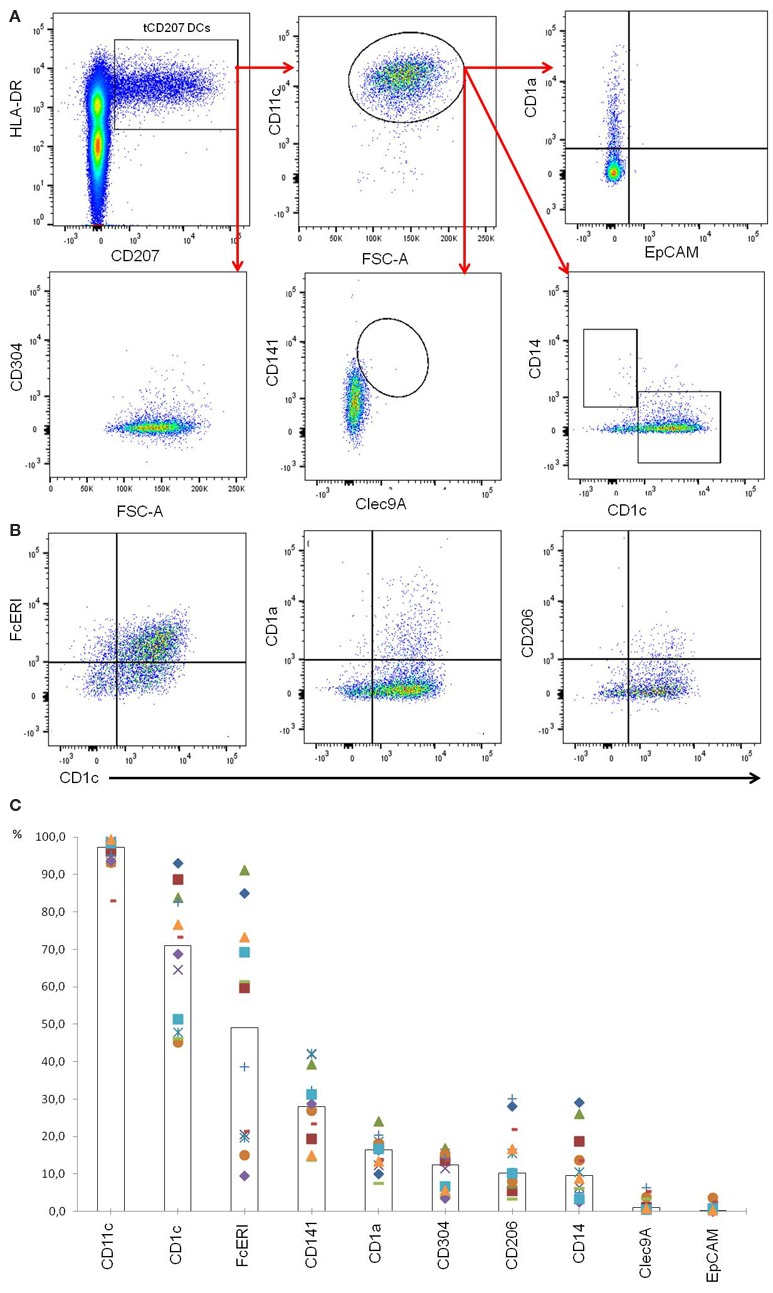
**Phenotypic characterization of tCD207 DCs**. **(A)** Dotplots represent tonsil mononuclear cells gated as lin^neg^ and analyzed for expression of HLA-DR, CD207, CD11c, CD1a, EpCAM, CD141, Clec9A, CD1c, CD14, and CD304. This analysis is representative of 12 independent experiments (10 children and 2 adults). **(B)** Dotplots represent tCD207 DCs gated as shown in **(A)** (lin^neg^HLA-DR^+^CD207^+^ cells) and analyzed for expression of CD1c, CD206, FcERI, and CD1a. **(C)** The graph shows the percent (%) of tCD207 DCs (lin^neg^HLA-DR^+^CD207^+^ cells) expressing CD11c, CD1c, FcERI, CD141, CD1a, CD304, CD206, CD14, Clec9A, and EpCAM. The medians of 12 independent experiments are represented by columns. All experiments are shown in distinct symbols.

Localization of tCD207 DCs in tonsils was investigated by IHC analysis performed on tissue sections (Figures 2A–F). The tCD207 DCs showed a typical DC morphology (Figure 2F) and were mainly detected outside germinal centers (GC) within the tonsillar lymphoid stroma (Figures 2A,B) but were also detected in tonsil epithelium (Figures 2C,D). Random counting of tCD207 DCs in tissue sections indicated a rather good correlation with the tCD207 DC frequency measured by FACS. We first investigated the possibility that tCD207 DCs may share some phenotypes with conventional LCs. FACS analysis of usual markers of conventional LCs such as CD11c, CD1a, and the epithelial cell adhesion molecule (EpCAM) revealed that while most tCD207 DCs expressed CD11c (median 97.3%), only a few tCD207 DCs expressed CD1a (median 16.5%), and no tCD207 DCs were found to express EpCAM (median 0.2%) (Figures 3A,C). Taking into account the atypical location of tCD207 DCs in tonsillar lymphoid stroma and the lack of expression of major markers of the LC lineage, we concluded that tCD207 DCs were clearly distinguishable from conventional LCs. Whether or not the few tCD207 DCs located within the tonsil epithelium represent true tonsillar LCs may require further investigation. Second, we also excluded the possibility that tCD207 DCs, which were initially described in viral inflammatory condition (12), may be related to human infDCs (18, 19) since major hallmarks of infDCs were detected only at a low frequency on tCD207 DCs (Figure 3), notably CD1a (median 16.5%), CD14 (median 9.6%), and CD206 (median 10.2%). Further analysis of cell surface expression of several DC markers (Figure 3) also showed that a high proportion of tCD207 DCs expressed CD1c (median 71%), but in contrast these DCs appeared distinct from others DC subsets like pDCs (HLA-DR^+^ CD304^+^), CD141/Clec9A DCs and CD14^+^ macrophages. Of note, a variable proportion of tCD207 DCs were found to express FcERI (median 49.2%), and a smaller proportion of these cells expressed CD141 (median 28.1%).

**Figure 4:**
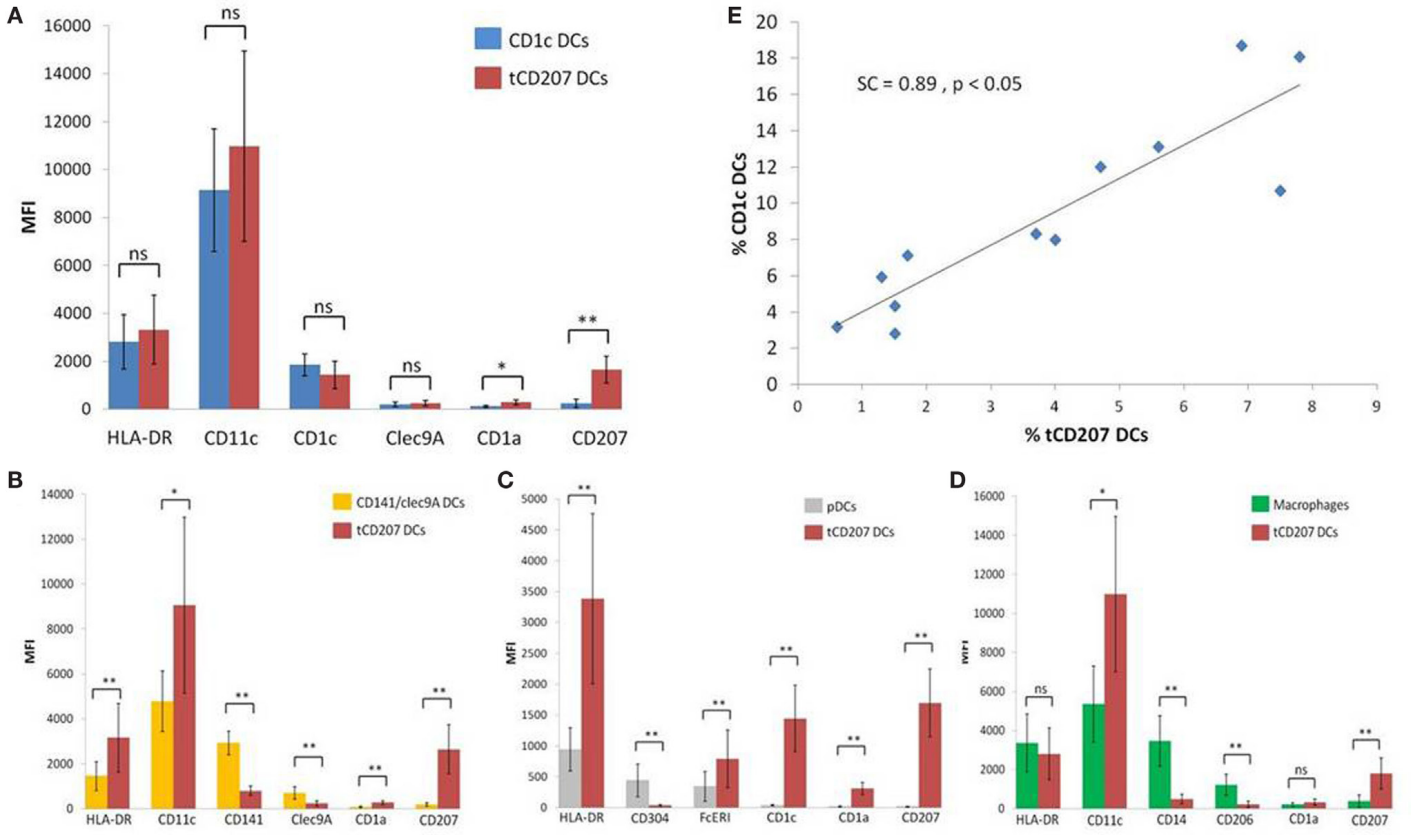
**Comparative analysis of levels of expression of different DC markers on tCD207 and other DC subsets**. **(A–D)** Graphs show the expression levels (MFI) of different indicated DC markers on tCD207 DCs (lin^neg^HLA-DR^+^CD207^+^) compared to **(A)** CD1c DCs (HLADR^+^CD11c^+^CD1c^+^), **(B)** CD141 DCs (HLADR^+^CD11c^+^CD141^+^Clec9A^+^), **(C)** pDCs (HLADR^+^CD304^+^), or **(D)** macrophages (HLADR^+^CD11c^+^CD14^+^). **(A–D)** Data are expressed as mean MFI values ± SD for 12 independent tonsil samples (10 children and 2 adults). *p* value comparing two groups (Mann–Whitney *U* test): *p < 0.05; **p < 0.01; ns: not significant. **(E)** Correlation between frequencies (%) of tCD207 DCs and CD1c DCs in 12 independent tonsils (10 children and 2 adults). Correlation coefficient of Spearman (SC) and *p* value are shown.

Last, we considered the expression level [mean fluorescence intensity (MFI)] of each DC marker to further characterize tCD207 DCs in comparison with other tonsillar DC subsets (Figure 4). The MFI of CD207 was significantly higher in tCD207 DCs compared with all other tonsillar HLA-DR^+^ cells. Interestingly, CD1c DCs and tCD207 DCs appeared to be closely related since, apart from CD207, no other significant difference was measured regarding levels of expression of typical CD1c DC markers. In particular, CD11c expression was high and CD1c was expressed at a similar level in both subsets while the expression of Clec9A was null (Figure 4A). In contrast, tCD207 DCs clearly differed from all other tonsillar DC subsets, namely, CD141/Clec9A DCs (Figure 4B), pDCs (Figure 4C), and macrophages (Figure 4D), since tCD207 DCs did not express most of the DC markers that constitute their conventional hallmarks. Collectively, these results showed that tCD207 DCs were probably not related to LCs, infDCs, CD141/Clec94 DCs, or monocytes/macrophages, but likely linked to the CD1c myeloid DC lineage through the expression of CD11c and CD1c and variable expression of FcERI and CD141. Moreover, the link between tCD207 DCs and CD1c DCs tonsillar population was also strengthened by the fact that the abundance of tCD207 DCs and CD1c DCs in each tonsil showed a strong positive linear correlation (SC = +0.897, *p* < 0.05, *n* = 12) (Figure 4E), while no significant correlation could be measured with all the other DC subsets (not shown).

## Discussion

This work follows up our previous work (12) describing for the first time a subpopulation of human DCs in adult tonsils with expression of CD207 while not being classical LCs. Better characterization of subpopulations of human DCs is timely and important since this cell type is a potential target for cell-based immunotherapy. We aimed here to present a detailed phenotypic characterization for defining the cell surface phenotype of the langerin/CD207-expressing DCs encountered in human tonsils human. Our results show that all human tonsils are consistently infiltrated with significant amount of tCD207 DCs that may represent around 3.2 and 7.3% of the total content of the HLA-DR^+^ tonsillar DCs in child and adult tonsils, respectively. Interestingly, these tCD207 DCs are also shown to be closely related to the myeloid CD1c DC lineage.

It was first tempting to speculate that the tCD207 DCs, which were initially discovered in an inflammatory viral environment (12), might share some properties with the human infDCs. Indeed, infDCs found in human inflammatory fluids, also have a relatively high level of CD207 gene expression compared to inflammatory macrophages (NCBI, Gene Expression Omnibus, Query DataSets for GSE40484). Although tCD207 DCs and infDCs have several conventional markers of the myeloid DC lineage (CD11c, CD1c, and FcERI) in common, we however exclude the possibility that tCD207 DCs could be related to infDCs since tCD207 DCs do not express CD1a, CD14, and CD206 which are consistent hallmarks of infDCS (18). Second, the CD207 marker was long believed to be exclusively expressed by LCs, which represent a distinct lineage from pDCs and cDCs (7), and represent major APCs within the epidermis and stratified epithelium of bronchi and mucosae (14, 15, 20–23). LCs are identified based on the expression of HLA-DR, CD207 but also high level of CD1a and EpCAM (15). The lack of hallmarks of the LC lineage (CD1a and EpCAM) coupled with the unusual location of CD207 DCs in tonsillar lymphoid stroma led us to conclude that tCD207 DCs are different from conventional epidermal LCs. In mouse, elegant works have established that dermal CD207^+^ DCs and LCs can represent distinct lineages (24–26). It was notably shown that the mouse dermis contains a population of CD207^+^ DCs, termed dermal CD207^+^ DCs, that develop independently of epidermal LC population (24–26). In human, the existence of CD207^+^ DCs unrelated to LCs has been proposed but is yet poorly described. The CD207 expression has been reported in the lamina propria of patients suffering of inflammatory bowel diseases (27) or celiac disease (28), in kidneys (29), or in breast cancer (30), but it remains to be determined whether these cells are related to LCs or not. Moreover, Bigley et al. (17) have recently reported the existence of human CD207^+^ DCs widely distributed and closely related to CD1c DCs but clearly distinguishable from LCs. In that study, CD1a^+^CD1c^+^CD207^+^ DCs were found in human dermis, while in lung, liver, and tonsil, CD1c^+^ DCs were shown to also express CD207 with a variable CD1a expression (17). Strikingly, the CD1c^+^CD207^+^ DCs described by Bigley and co-workers (17) display a very similar profile to the tCD207 DCs we described here, with a high level expression of CD11c and CD1c, and the lack of characteristic markers of CD141 DCs such as Clec9A (17). Moreover, we show here that tCD207 DCs in children and adults are present in lymphoid stroma and not only in tonsil epithelium but also these cells are also related to CD1c DCs with variable FcERI and intermediate CD141 expression. In addition, we observed a significant correlation in the abundance of tCD207 DCs and CD1c DCs in tonsil, suggesting a direct relationship between these two types of DCs. Collectively, these observations thus strongly suggest that the tCD207 DCs we describe here are likely closely related to CD1c resident DCs and may belong to this DC subset. Since it was established that CD1c myeloid DCs exhibit plasticity and are capable of expressing CD207 notably in the presence of inflammatory cytokines like TGFβ (17, 31, 32), an attractive hypothesis could be that some resident tonsillar CD1c DCs may express CD207 under local inflammatory conditions. One caveat of our experiments is that all tonsils used in this study were from patients with chronic tonsillitis or tonsillar hypertrophy. The fact that these pathological conditions may be responsible for CD207 expression in CD1c DCs is an interesting question. Notably it would be interesting to determine whether the tCD207 DC population shares similar gene expression signatures and functional properties with the CD1c DC subset. In particular, CD1c DCs are competent to activate CD4 T cells and can induce Th1 or Th17 polarization but are poor inducers of Th2 responses (7). Whether tCD207^+^CD1c^+^ DCs play a similar role, or may likely play specific activities that differ from conventional CD1c DCs will be investigated.

The selective expression of pattern recognition receptors, including lectins and TLRs, confers specialized functions to different populations of DCs. Langerin/CD207 is known to bind pathogens like mycobacteria (33), fungi species (34), viruses such as HIV (35), influenza (36), or HSV (37), but the function of CD207 in antigen uptake is still unclear. The role of DCs expressing CD207 as inducers of immunity (38–40), as tolerogenic modulators (28, 41–43) or as antigen transporters between different DCs (44), is debated. Interestingly, we previously discovered tCD207 DCs in adult tonsils infected with EBV and showed that these cells were geographically grouped in close contact with tonsillar EBV-infected cells suggesting a possible link between EBV infection and tonsillar DC infiltration. To further investigate a possible link between EBV infection and tCD207 DC, we focused here on the analysis of tonsils collected from children known to be infected or naïve regarding EBV infection. Indeed, EBV infection is highly prevalent in the human adult population (95%) and usually occurs early during infancy (45). Using tonsils from young children, we were here able to demonstrate that tCD207 DCs were present in tonsils independently of the individual’s EBV status. This result indicates that tCD207 DCs are present in tonsils even before EBV-infection. Although this result may appear to be contradictory with our previous observations achieved in adult tonsils, it must be pointed out that adult and child tonsils may differ greatly, not only regarding tonsil structure and immune functions, but also concerning local inflammatory conditions associated with long-term recurrent tonsillitis. One hypothesis could be that long-term chronic EBV replication in adult tonsils may worsen local inflammation and may favor the persistence of tCD207 DCs, mainly in EBV-infected clusters (12). In this context, the higher tCD207 DC frequency observed in EBV-infected adult tonsils may strengthen this hypothesis. Previously, we also found abundant CD207-expressing DCs in EBV-associated malignancies (12). Whether these CD207-expressing DCs found in EBV-associated tumors are similar to tCD207 DCs and what role these DCs may play in tumor biology is of great interest and remains to be further investigated.

## Author Contributions

AM, CO, JM, ES, and AD designed the research. AM, CO, and SV performed the research. AM, CO, VG, JM, ES, and AD contributed to data analyses and data interpretation. SB and LC contributed to clinical information and samples for the study. AM, JM, ES, and AD wrote the manuscript.

## Conflict of Interest Statement

The authors declare that the research was conducted in the absence of any commercial or financial relationships that could be construed as a potential conflict of interest.
